# *Rhinolophus sinicus* virome revealed multiple novel mosquito-borne zoonotic viruses

**DOI:** 10.3389/fcimb.2022.960507

**Published:** 2022-10-11

**Authors:** Chengcheng Peng, Duo Zhang, Chenghui Li, Yiquan Li, He Zhang, Nan Li, Pengpeng Xiao

**Affiliations:** ^1^ Wenzhou Key Laboratory for Virology and Immunology, Institute of Virology, Wenzhou University, Wenzhou, China; ^2^ College of Agriculture, Yanbian University, Yanji, China; ^3^ Academician Workstation of Jilin Province, Changchun University of Chinese Medicine, Changchun, China; ^4^ Changchun Veterinary Research Institute, Chinese Academy of Agricultural Sciences, Changchun, China

**Keywords:** metavirome analysis, bat, virome, viral isolation and identification, phylogenetic analysis

## Abstract

To exploit the *Rhinolophus sinicus*–specific virome, 29 *Rhinolophus sinicus* were gathered in Lincang, China. Enriched viral sequences of 22 virus families were acquired by metavirome techniques. Hereby, the part of virome in *Rhinolophus sinicus*, including Chikungunya virus (CHIKV), Getah virus, and Japanese encephalitis virus (JEV) were validated by PCR. Five CHIKV viral sequences were amplified, among which CHIKV-China/B2016C-1 shared the highest homology to CHIKV isolated from Italy in 2007, with the genotype as African ECS. Eight JEV viral sequences were amplified, of which JEV-China/B2016E-1 shared the highest homology with at least 91.3% nt identity with the JEV sequence found in South Korea in 1988 and was classified as genotype III. Notably, JEV was isolated for the first time in *Rhinolophus sinicus*. The newly isolated JEV-China/B2016-1 could increase infectivity while passaging in Vero cells from BHK-21 cells. Overall, the research sheds insight into the diversity and viral susceptibility dynamics of the virome in *Rhinolophus sinicus* and reveals new light on the ecology of other important viral hosts.

## Introduction

Since the SARS-CoV-2 outbreak, research on viruses carried by bats has increased dramatically, and a large number of novel viruses have been isolated or detected from bats (Laura et al., 2022; Gábor et al., 2022; Kate and Edward, 2022). Because bats have no obvious clinical signs of virus infection, they are considered to be the natural hosts of many zoonotic viruses (Natalia et al., 2018). Yunnan province of China is located at the border of four countries (China, Myanmar, Laos, and Vietnam) (Chao et al., 2017; Rui-Chen et al., 2020), where viruses carried by bats can easily cause cross-border transmission. In particular, *Rhinolophus sinicus* is the common bat species in this area (Susanna et al., 2017). Thus, virus monitoring for the specific bat serves important research utility. To verify accurately limited number of virus existence, polymerase chain reaction (PCR) technology and Western blot could be more useful (Micah et al., 2021; Ptasinska et al., 2021). Nevertheless, metagenomic sequencing has shown strong strength for the discovery of a vast variety of low-abundance and unknown viruses (Jenai et al., 2019). The rapid development of metagenomic sequencing has led to a spurt in virus identification (Haoming et al., 2020). Hence, applying metagenomic sequencing to bats can be used not only for the mining of novel susceptible viruses but also effectively prevent the underdetection of unknown viruses at low abundance.

To better assess the potential hazards posed by the specific bat to human health, we conducted the research to exploit *Rhinolophus sinicus*-specific viruses in Yunnan province and to present a precious resource for the diagnosis, isolation and identification of novel susceptible viruses. A rich variety of viral species, including novel susceptible viruses, were identified in *Rhinolophus sinicus* from Yunnan province by metavirome techniques. In addition to new variants of CHIKV and Getah virus, Japanese encephalitis virus (JEV) was isolated for the first time from *Rhinolophus sinicus*. The initial research on the virome of *Rhinolophus sinicus* yield valuable insights into the identification, diversity, and surveillance of the virome of other bat species.

## Material and methods

### Bat collection

**Figure 1:**
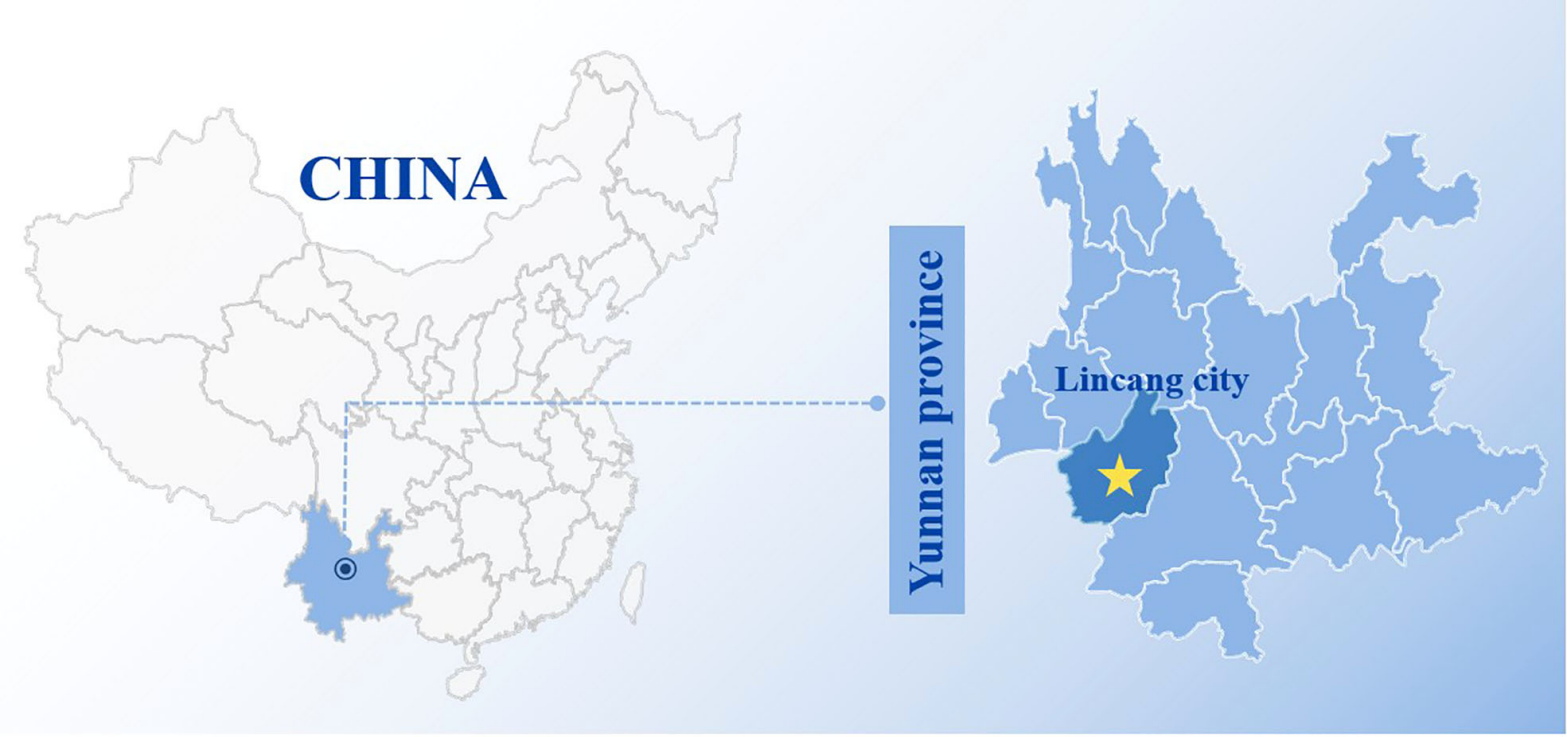
The sample collection site in Yunnan province, China. The sample collection site was labeled with yellow solid pentacle.

Bats were sampled in natural habitat caves using mist nets from Lincang city (N 23° 89’, E 100° 09’) in Yunnan province (**Figure 1**) from June to July 2016. Briefly, the bats at the top of natural habitat caves were stimulated by the flashlight light and fell into the pre-arranged mist nets. The trapped bats were retrieved from mist nets using anti-bite gloves. Cloth bags were used to individually confine and transport bats to laboratory facilities for processing. After morphological identification and Cytb gene identification, all throat swabs were collected from *Rhinolophus sinicus*. All throat swabs were placed into 2-ml polyethylene tubes with swab preservation solution and temporarily stored at −80°C. The studies involving animals were reviewed and approved by Wenzhou University Laboratory Animal Welfare and Ethics Committee.

### RNA extraction and metaviral sequencing

The reagents, RNA extraction methods, and metaviral sequencing have been described in our previous study (Guanrong et al., 2022). The DNA barcodes used for metaviral sequencing was shown in **Table 1**. Blastn (https://blast.ncbi.nlm.nih.gov/Blast.cgi) was performed for contigs comparison with GenBank, where *E* value ≤ 10e^-5^ was included in the adoption to identify viral sequences. Application of RT-PCR using specific primers to confirm viral sequences and further validate metaviral sequencing results (**Table 2**).

**Table 1:** Barcode DNA employed in metagenomic analysis (Pengpeng et al., 2018).

Primer type	Primer number	Primers (5′-3′)
Anchored Random Primers	RT1	GCCGGAGCTCTGCAGATATCNNNNNN
	RT 2	GTATCGCTGGACACTGGACCNNNNNN
	RT 3	ATCGTCGTCGTAGGCTGCTCNNNNNN
Barcode Primers	Primer1	GCCGGAGCTCTGCAGATATC
	Primer2	GTATCGCTGGACACTGGACC
	Primer3	ATCGTCGTCGTAGGCTGCTC

**Table 2:** Primer pairs used in PCR identification.

Primer name	Primers (5′-3′)	Product (bp)
CHIKV-China/B2016C-1/2/3-F	ATGGAGTTCATCCCAACCCA	783
CHIKV-China/B2016C-1/2/3-R	CCACTCTTCGGCCCCCT	
CHIKV-China/B2016NS1-1/2-F	ATGGATCCTGTGTACGTGCACA	1605
CHIKV-China/B2016NS1-1/2-R	TGCGCCCGCTCTGTCCTCAA	
GETV-China/B2016E2-1/2-F	AGTGTGACGGAACACTTCAAT	1266
GETV-China/B2016E2-1/2-R	GGCATGCGCTCGTGGTGC	
JEV-China/B2016E-1/2/3/4-F	TTTAATTGTCTGGGAATGGG	1500
JEV-China/B2016E-1/2/3/4-R	AGCATGCACATTGGTCGCTA	
JEV-China/B2016NS1-1/2/3/4-F	GACACTGGATGTGCCATTGAC	1056
JEV-China/B2016NS1-1/2/3/4-R	AGCATCAACCTGTGATCTAAC	
JEV-F*	ATCTGTCACTAGATCGGAGCA	359
JEV-R*	CGTCAGTGCTCTCCTCTCTA	

*The primers used in JEV identification after viral isolation.

### Phylogenetic analysis

The amplified complete viral genes were compared with representative viral genes by Clustal W 2.0. Complete C gene sequences with 783 nt of CHIKV, E2 gene sequences with 1266 nt of GETV, and E gene sequences with 1500 nt of JEV were used for phylogenetic analysis. Evolutionary tree model was constructed by the maximum-likelihood algorithm with bootstrap value set to 1,000 using MEGA 7.0.

### Virus isolation and identification

For the isolation of virus in BHK-21 and Vero cells, cells were cultured in medium (DMEM, HyClone, Logan, UT, USA) containing 8% fetal bovine serum. Briefly, bat sample grinding solutions were centrifuged, and 20 μl of supernatant was added to BHK-21 cells, incubated at 37°C for 5–7 days until virus-induced cytopathic effects (CPEs) were observed. JEV in the supernatant was detected using JEV-NS1 specific primers (**Table 2**). The expression of NS1 protein was tested through Western blotting with the anti-NS1 monoclonal antibody (Abcam, Cambridge, UK) and an HRP-conjugated rabbit anti-mouse antibody (Trans, Beijing, China). Negative-stained electron microscopy was employed for the observation of JEV particles, which were prepared using supernatant of infected BHK-21 cells mingled 1:1 with 6.1% paraformaldehyde, mounted on copper grids, with the treatment of 3% phosphotungstic acid (Duo et al., 2022).

### Viral passage and variation analysis

Newly isolated JEV were trans-species passaged into Vero cells and subsequently mimic natural infection at the cellular level. Specifically, JEV was first passaged to the 10^th^ generation in BHK cells, then virus was extracted from the supernatant and inoculated into Vero cells for 10 passages, with a total of 20 passages. The TCID_50_ in BHK cells of 5^th^ and 10^th^ generation viruses and Vero cells of 15^th^ and 20^th^ generation was detected by Reed-Muench method (Krah, 1991). Subsequently, the NS1 gene of the above four passages were sequenced and compared using MEGA 7.0.

### Statistical analysis

Statistics were performed using SPSS 26.0 software. *t*-test was used in the comparison among different groups after normality test. Each experiment was validated iteratively by setting up three replicates and repeating it three times. *P* < 0.05 was considered statistical significance.

### Data availability

The data produced in metagenomic sequencing were registered in the GenBank (Accession No. SRR15292018). The amplified viral sequences have been deposited in the GenBank, in which the accession numbers were covered: CHIKV-China/B2016C-1/2/3 (OM799585–OM799587), CHIKV-China/B2016NS1-1/2 (OM799577–OM799578), GETV-China/B2016E2-1/2 (OM799568–OM799569), JEV-China/B2016E-1/2/3/4 (OM799548–OM799551), and JEV-China/B2016NS1-1/2/3/4 (OM799558–OM799561), respectively.

## Results

### Bat virome

**Figure 2:**
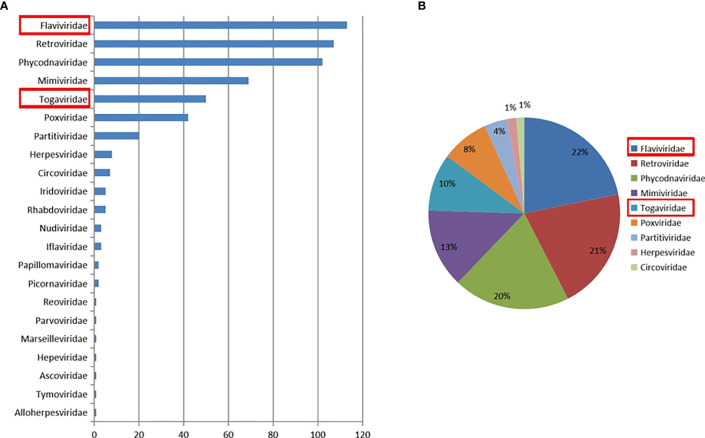
Abundance and proportion of viral families in *Rhinolophus sinicus*. The taxonomy of viral sequences according to viral family, with relative abundance **(A)**. The proportion of main viral families in *Rhinolophus sinicus* **(B)**.

A total of 29 throat swabs from 29 bats classified as *Rhinolophus sinicus* were sampled for metaviral sequencing. Sequences were sorted as viruses with 3.67 × 10^7^ reads (average length of 186.52 nt), including known and unclassified viruses. Numerous bat viruses and unknown or unclassified viruses have been identified with potential risk of infection to humans and animals. Abundance of virus reads varied from different viral taxonomy. A total of 22 viral families were detected (**Figure 2**), of which *Flaviviridae* and *Togaviridae*, consisted of known vital human pathogens verified in *Rhinolophus sinicus*. Metavirome assay results showed that *Rhinolophus sinicus* carried numerous potential zoonotic viruses. A total of 40,351 contigs were yielded from *dDe novo* assembly. Several attractive assembled contigs displayed similarities to viral sequences of *Flaviviridae* and *Togaviridae*, among which 37 GETV-like contigs, 84 CHIKV-like contigs, and 206 JEV-like contigs demonstrated reads coverage of 27× (223–816 nt), 32× (238–1035 nt) and 38× (255–1841 nt) respectively. In addition, the GETV-like contigs, CHIKV-like contigs and JEV-like contigs possessed 83.1–92.5%, 82.7–94.1%, and 84.0–93.6% nt homology with the known GETV, CHIKV, and JEV sequences separately. Afterward, the detected virus-like sequences was amplified with designed virus-specific primers (**Table 2**), and thus validated the metaviral sequencing results.

### The verification of CHIKV-like sequences

**Figure 3:**
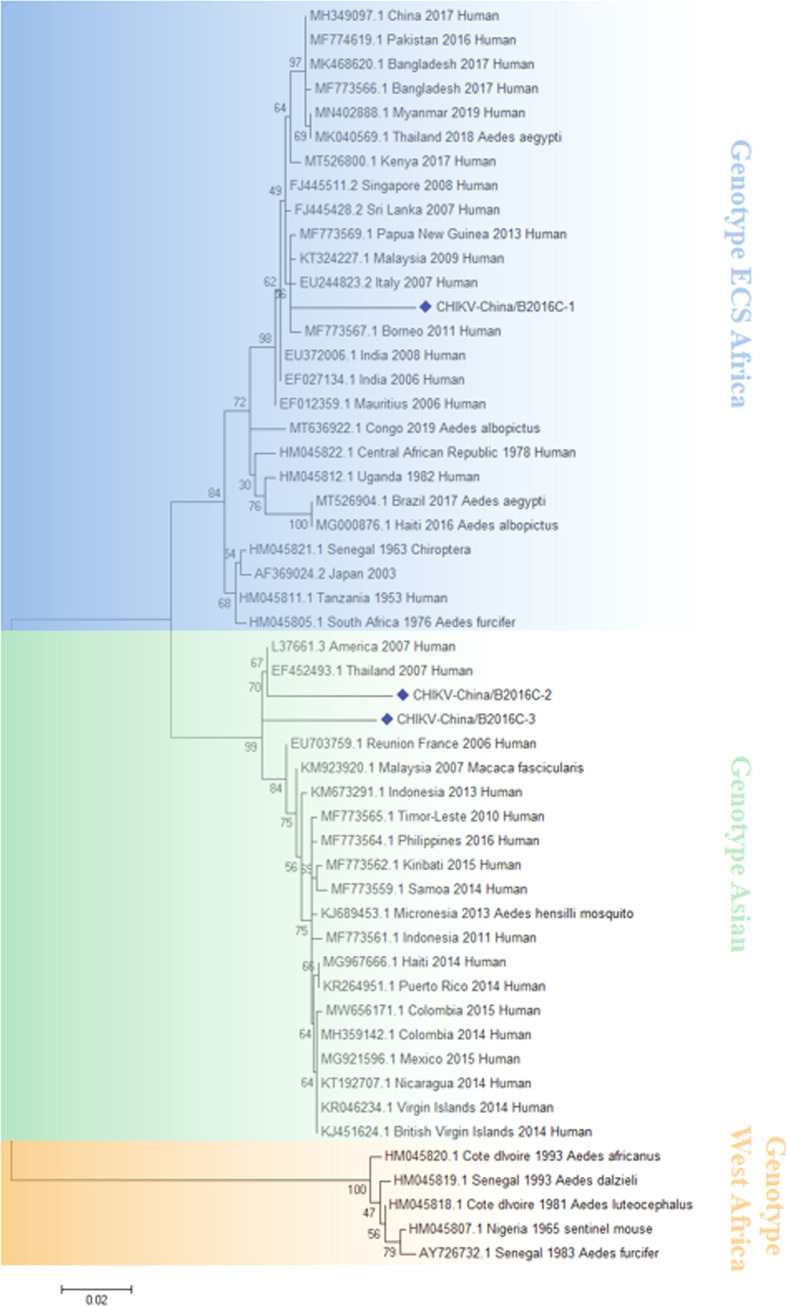
The phylogenetic analysis based on CHIKV-C. Complete C gene sequences with 783 nt of CHIKV were used for phylogenetic analysis. The maximum likelihood method was used tree building algorithms in MEGA 7.0, with bootstrap value set to 1,000. Solid blue rhombus was used for marking the viral genes amplified in the research.

The CHIKV-like fragments identified in this research were amplified by PCR, cloned into pMD19-T (Takara, Tokyo, Japan) and sequenced. The results showed that the sequence of the two NS1 genes were all 1605 nucleotides in length (CHIKV-China/B2016NS1-1/2), and the isolates shared ~95.8% nt homology and ~91.2% aa homology among them. Three 783-bp fragments (CHIKV-China/B2016C-1/2/3) were also obtained, and the similarities of nt and aa sequences of C genes among these isolates are 89.5–93.7% and 89.5–93.7%, respectively. Analysis using BLASTN indicated that CHIKV-China/B2016C-2/3 displayed the highest nt (96.7%) and aa (93.1%) homology with CHIKV from Thailand isolated in 2007, which was assigned to genotype Asian, whereas CHIKV-China/B2016C-1 had the highest homology with CHIKV from Italy isolated in 2007 (96.8% at nt and 92.3% at aa), which was assigned to genotype ECS Africa (**Figure 3**).

### The verification of GETV-like sequences

**Figure 4:**
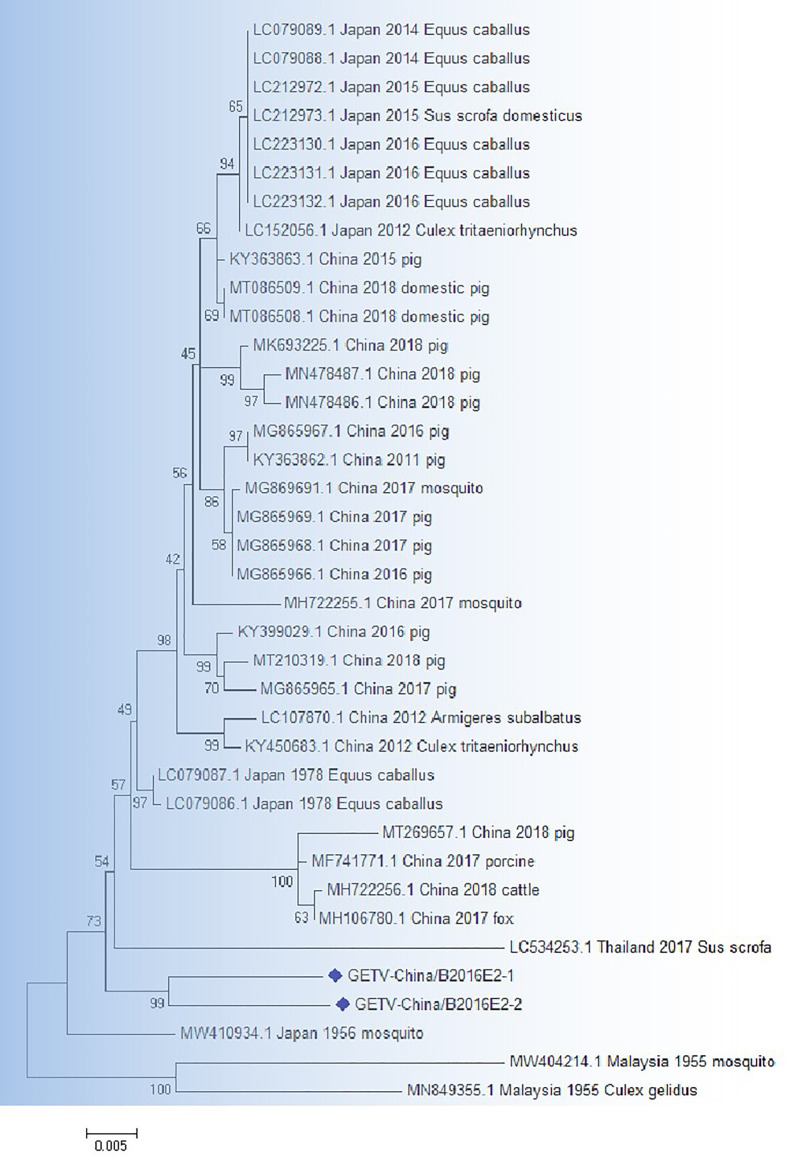
The phylogenetic analysis based on GETV-E2. Complete E2 gene sequences with 1266 nt of GETV were used for phylogenetic analysis. The maximum likelihood method was used tree building algorithms in MEGA 7.0, with bootstrap value set to 1,000. Solid blue rhombus was used for marking the viral genes amplified in the research.

RT-PCR product of the GETV-like fragments were cloned in pMD19-T vector (Takara, Tokyo, Japan) and then sequenced. Two 1266-bp fragments (GETV-China/B2016E2-1/2) were acquired. The identities of nn and aa sequences of E2 genes of isolates are ~96.9% and ~95.5%, respectively. BLASTN results showed that the amplified GETV-like fragments had highest homologous (94.2% at nt and 93.8% at aa) to the E2 gene of GETV from Thailand isolated in sus scrofa, 2017 (**Figure 4**).

### The verification of JEV-like sequences

**Figure 5:**
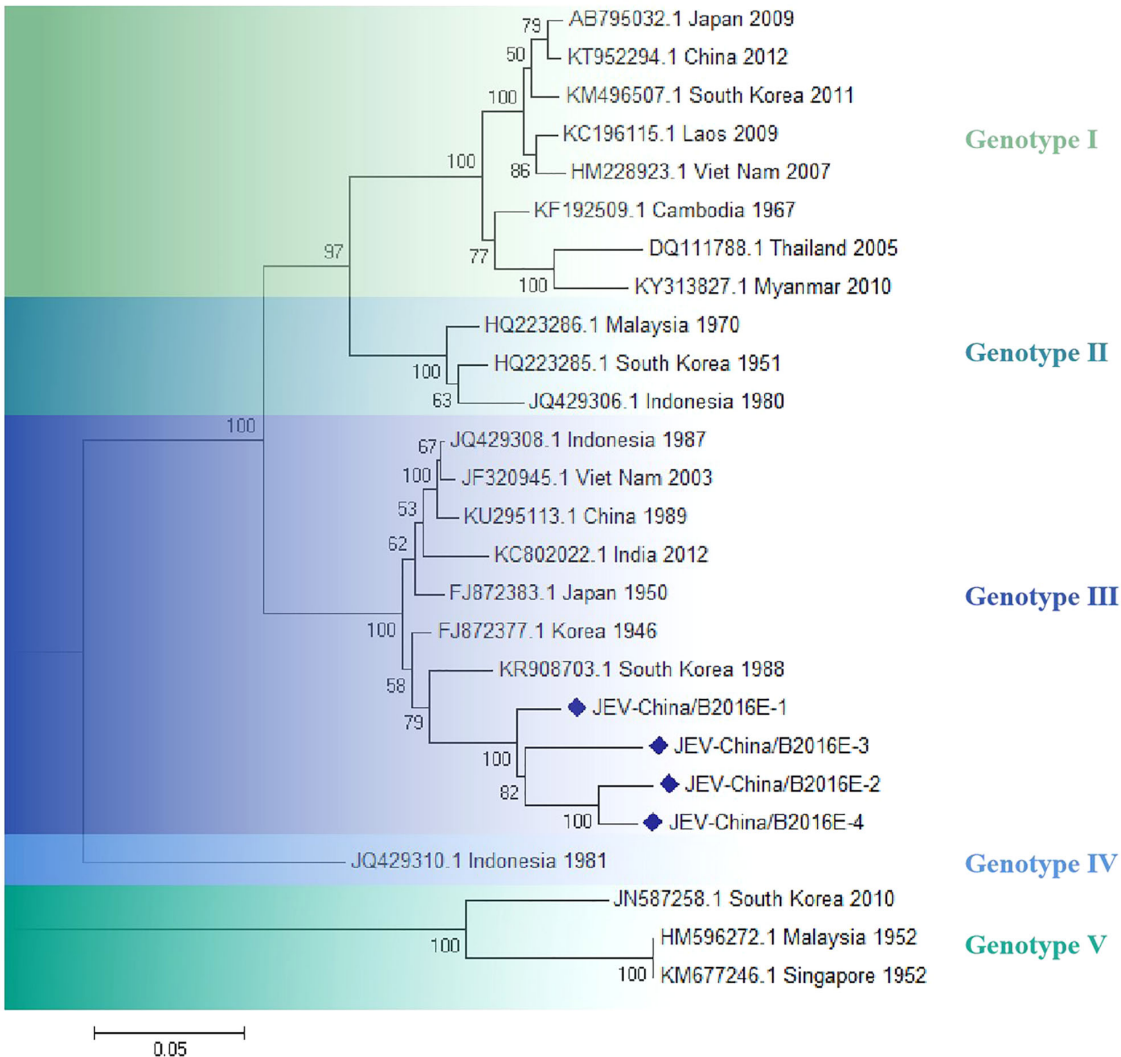
The phylogenetic analysis based on JEV-E. Complete E gene sequences with 1500 nt of JEV were used for phylogenetic analysis. The maximum likelihood method was used tree building algorithms in MEGA 7.0, with bootstrap value set to 1,000. Solid blue rhombus was used for marking the viral genes amplified in the research.

The amplified fragments of JEV-like gene were cloned in pMD19-T vector (Takara, Tokyo, Japan) and were sequenced. The sequencing analysis showed that there were four 1500-bp fragments (JEV-China/B2016E-1/2/3/4) and that identity of sequence was ~92.9–97.1% at nt and ~85.6–94.4% at aa. Moreover, the homologies were ~95.1–96.3% on nucleotide level and ~87.2–91.2% on amino acid level among the four fragments with 1056-bp (JEV-China/B2016NS1-1/2/3/4). Analysis using BLASTN indicated that JEV-like fragments were detected with the highest homologous (91.3% at nt and 85.6% at aa) to the JEV from South Korea isolated in 1988, which was assigned to genotype III JEV (**Figure 5**).

### Viral isolation identification of JEV

**Figure 6:**
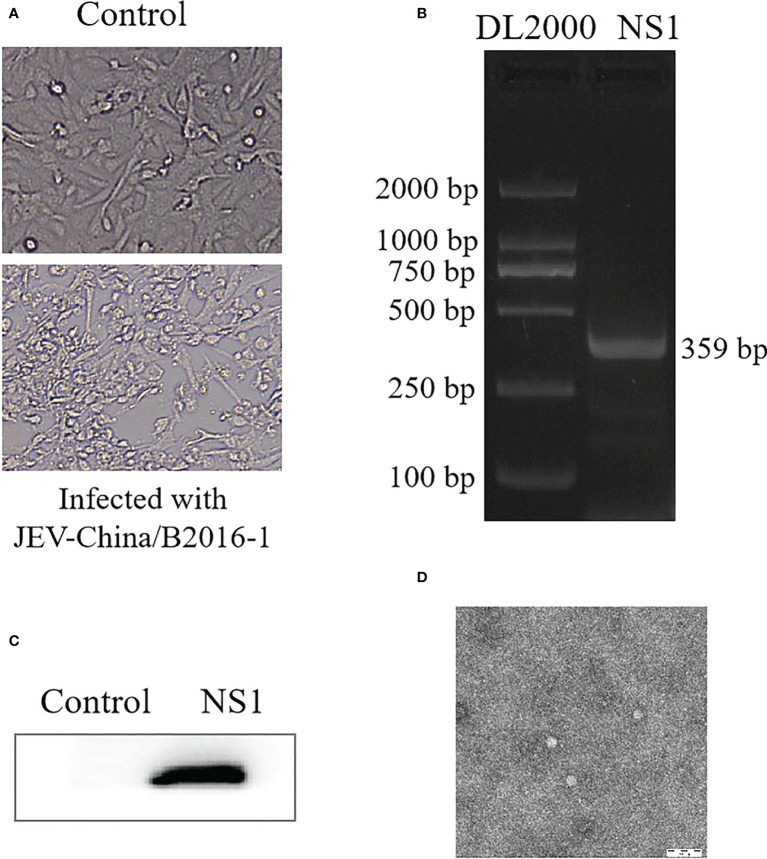
Identification of *JEV-China/B2016-1* isolated in Lincang city of Yunnan province. CPE observation of *JEV-China/B2016-1* after BHK-21 cells infection **(A)**. Identification of *JEV-China/B2016-1* by PCR **(B)**. Verification of *JEV-China/B2016-1* by Western blot **(C)**. Negative-stain electron microscopy of *JEV-China/B2016-1* particles **(D)**.

JEV was successfully isolated from the validated virus. The newly isolated JEV was verified by CPE (**Figure 6A**), PCR from the gene level (**Figure 6B**) and Western blotting from the protein level (**Figure 6C**), respectively, and all the results showed positive. We observed the virus particles by negative electron microscope, which is convenient for more intuitive verification of the newly isolated JEV. The virus showed a circular shape with a diameter of 30–40 nm and tiny protrusions on the surface (**Figure 6D**).

### The variability of JEV after consecutive passages

The infection titer value of JEV-China/B2016-1 in 5^th^ generation BHK cells was 5.26 × 10^3^ TCID_50_/0.1 ml, and that in 10^th^ generation was 4.67 × 10^3^ TCID_50_/0.1 ml. The infectivity was decreased in 10^th^ generation compared to 5^th^ generation, but the difference was not significant, *P* > 0.05 (*t*-test). Interestingly, in Vero cells, the infection titers of in generation 15^th^ and 20^th^ were 8.33 × 10^3^ TCID_50_/0.1 ml and 1.13 × 10^4^ TCID_50_/0.1 ml, respectively, both of which were significantly more infectious than those of BHK in generation 5^th^ and 10^th^, *P* < 0.05 (*t*-test). Therefore, it can be speculated that cross-species transmission may be one of the reasons for the increased infectivity of the virus. Further sequence analysis showed that the key amino acid site (E138D) of the E gene was mutated in both the 15^th^ and 20^th^ generations compared to the 5^th^ and 10^th^ generations.

## Discussion

Bats are the vectors and intermediate hosts of many zoonotic viruses, posing a major threat to human life and health (Smriti, 2022; Sarah et al., 2022; Ian et al., 2020). The *Rhinolophus sinicus* was a common bat species in Yunnan province, China (Hayes et al., 2019; Linmiao et al., 2022). Several studies have confirmed that *Rhinolophus sinicus* can transmit a variety of important viruses, covering SARS-CoV-2 (Hong-Liang et al., 2021), hepatitis B virus (Bornali et al., 2021), and bocaparvovirus (Susanna et al., 2016; Susanna et al., 2017). In our study, we conducted a preliminary exploration of the viral spectrum of *Rhinolophus sinicus* in Yunnan province employing metavirome technology and identified a rich set of viral sequences pertaining to 22 virus families. The research provides support for focusing on the breadth of the viral spectrum of common bat species circulating in this area. Moreover, it suggests the importance of conducting biological vector-specific surveillance.

According to the metavirome results, CHIKV and Getah viruses were simultaneously amplified from *Rhinolophus sinicus*, indicating that CHIKV and Getah viruses were in potential dissemination and prevalence in the same bat species and geographical location, existing a co-transmission probability. Interestingly, JEV was isolated successfully. To our knowledge, this is the first report of JEV isolation from *Rhinolophus sinicus*. This suggests that JEV has the potential to expand the species of susceptible bats and hence the geographic range of prevalence. The mechanisms underlying the species range of JEV-sensitive bats need further study.

Variability analysis of JEV showed that compared with the 5^th^ and 10^th^ generations, the E gene was mutated at a critical amino acid site (138) in generations 15 and 20. According to reports, E-138 mutation will significantly change the viral infectivity (Ni et al., 1994). In this study, compared with generations 5 and 10, both generations 15 and 20 showed a significant enhance in infectivity, implying that JEV may be able to transmit across species barriers, thereby enhancing infectivity.

Our research showed the potential viral spectrum in the *Rhinolophus sinicus* in the area. Nevertheless, the specific viral landscape of *Rhinolophus sinicus* is not well established. Due to the limitation of sample size and sampling range, there are many potential viruses that have not yet been excavated. The potential viruses of specific bat species need to be confirmed by further studies. In summary, our research offers precious insights into the spectrum of viruses propagated by specific bats and the discovery of their new susceptible viruses. It will serve as an essential reference value for molecular epidemiological studies of biological vectored viruses.

## Data availability statement

The datasets presented in this study can be found in online repositories. The names of the repository/repositories and accession number(s) can be found in the article/**Supplementary Material**.

## Ethics statement

The animal study was reviewed and approved by Wenzhou University Laboratory Animal Welfare and Ethics Committee.

## Author contributions

PX and NL conceived and designed the experiments. CP, DZ, CL, and HZ performed the experiments. PX and YL analyzed the data. CL contributed reagents/materials/analysis tools. PX and NL wrote the paper. All authors read and approved the final version of the manuscript.

## Funding

This work was supported by the National Natural Science Foundation of China (grant number 32002312), the Natural Science Foundation of Zhejiang Province (grant number LQ21H160001), Science and Technology Project of Wenzhou, Zhejiang, China (grant number Y20210080 and Y2020103).

## Conflict of interest

The authors declare that the research was conducted in the absence of any commercial or financial relationships that could be construed as a potential conflict of interest.

## Publisher’s note

All claims expressed in this article are solely those of the authors and do not necessarily represent those of their affiliated organizations, or those of the publisher, the editors and the reviewers. Any product that may be evaluated in this article, or claim that may be made by its manufacturer, is not guaranteed or endorsed by the publisher.

## References

[B1] Bornali D. Arif U. Supriyo C. (2021). Analysis of codon usage of horseshoe bat hepatitis b virus and its host. Virology. 561, 69–79. doi: 10.1016/j.virol.2021.05.008 34171764

[B2] Chao W. Xiaofang G. Jun Z. Quan L. Hongbin L. Edward B. M.. (2017). Behaviors related to mosquito-borne diseases among different ethnic minority groups along the China-Laos border areas. Int. J. Environ. Res. Public. Health 14 (10), 1227. doi: 10.3390/ijerph14101227 PMC5664728 29036937

[B3] Duo Z. Chengcheng P. Chenghui L. Yiquan L. He Z. Nan L.. (2022). Metavirome analysis of culextritaeniorhynchus reveals novel Japanese encephalitis virus and chikungunya virus. Front. Cell. Infect. Microbiol. 12. doi: 10.3389/fcimb.2022 PMC9280054 35846772

[B4] Gábor K. Gábor E. T. Martin M. Simon S. Nigel T. Edward W.. (2022). Isolation of infectious lloviu virus from schreiber's bats in Hungary. Nat. Commun. 13 (1), 1706. doi: 10.1038/s41467-022-29298-1 35361761 PMC8971391

[B5] Guanrong F. Jinyong Z. Ying Z. Chenghui L. Duo Z. Yiquan L.. (2022). Metagenomic analysis of togaviridae in mosquito viromes isolated from yunnan province in China reveals genes from chikungunya and Ross river viruses. Front. Cell. Infect. Microbiol. 12. doi: 10.3389/fcimb.2022.849662 PMC8878809 35223559

[B6] Haoming W. Rui P. Tong C. Liang X. Haiyan Z. Tao L.. (2020). Abundant and diverse RNA viruses in insects revealed by RNA-seq analysis: Ecological and evolutionary implications. mSystems. 5 (4), e00039–e00020. doi: 10.1128/mSystems.00039-20 32636338 PMC7343303

[B7] Hayes K. H. L. Xin L. Joshua F. Susanna K. P. L. Patrick C. Y. W. (2019). Molecular epidemiology, evolution and phylogeny of SARS coronavirus. Infect. Genet. Evol. 71, 21–30. doi: 10.1016/j.meegid.2019.03.001 30844511 PMC7106202

[B8] Hong-Liang Z. Yu-Ming L. Jing S. Yu-Yuan Z. Tong-Yun W. Ming-Xia S.. (2021). Evaluating angiotensin-converting enzyme 2-mediated SARS-CoV-2 entry across species. J. Biol. Chem. 296, 100435. doi: 10.1016/j.jbc.2021.100435 33610551 PMC7892319

[B9] Ian N. B. Elaine X. Katrina B. M. Pamela C. D. L. C-R. Jennifer L. E. Benjamin M.. (2020). RTP4 is a potent IFN-inducible anti-flavivirus effector engaged in a host-virus arms race in bats and other mammals. Cell. Host. Microbe 28 (5), 712–723. doi: 10.1016/j.chom.2020.09.014 33113352 PMC7666060

[B10] Jenai Q. Charles L. Alison K. Joshua B. Noam T. Amy L.. (2019). FLASH: a next-generation CRISPR diagnostic for multiplexed detection of antimicrobial resistance sequences. Nucleic. Acids Res. 47 (14), e83. doi: 10.1093/nar/gkz418 31114866 PMC6698650

[B11] Kate V. B. Edward C. H. (2022). Zoonotic disease and virome diversity in bats. Curr. Opin. Virol. 52, 192–202. doi: 10.1016/j.coviro.2021.12.008 34954661 PMC8696223

[B12] Krah D. L. (1991). A simplified multiwell plate assay for the measurement of hepatitis a virus infectivity. Biologicals. 19, 223–227. doi: 10.1016/1045-1056(91)90039-M 1659431

[B13] Laura A. P. Alison J. P. Karrie R. Justin A. W. Michelle L. B. Victoria B. (2022). Serological evidence of a pararubulavirus and a betacoronavirus in the geographically isolated Christmas island flying-fox (Pteropus natalis). transbound. Emerg. Dis. 69(5):e2366–e2377. doi: 10.1111/tbed.14579 PMC9529767 35491954

[B14] Linmiao L. Libiao Z. Jiabin Z. Xiangyang H. Yepin Y. Ping L.. (2022). Epidemiology and genomic characterization of two novel SARS-related coronaviruses in horseshoe bats from guangdong. China. mBio. 13(3) e0046322. doi: 10.1128/mbio.00463-22 35467426 PMC9239062

[B15] Micah T. M. Florica J. C. Bradly P. N. Marshall N. Thomas W. B. Ricardo H.. (2021). A blood-based host gene expression assay for early detection of respiratory viral infection: an index-cluster prospective cohort study. Lancet Infect. Dis. 21 (3), 396–404. doi: 10.1016/S1473-3099(20)30486-2 32979932 PMC7515566

[B16] Natalia L. F. Elaine R. F. Sandriana D. R. S. Fernanda G. L. Orlando G. R. Iana S. S. K. (2018). Street Rabies virus strains associated with insectivorous bats are less pathogenic than strains isolated from other reservoirs. Antiviral. Res. 160, 94–100. doi: 10.1016/j.antiviral.2018.10.023 30393124

[B17] Ni H. Burns N. J. Chang G. J. Zhang M. J. Wills M. R. Trent D. W.. (1994). Comparison of nucleotide and deduced amino acid sequence of the 5’ non-coding region and structural protein genes of the wild-type Japanese encephalitis virus strain SA14 and its attenuated vaccine derivatives. J. Gen. Virol. 75, 1505–1510. doi: 10.1099/0022-1317-75-6-1505 8207417

[B18] Pengpeng X. Chenghui L. Ying Z. Jicheng H. Xiaofang G. Lv X.. (2018). Metagenomic sequencing from mosquitoes in China reveals a variety of insect and human viruses. Front. Cell. Infect. Microbiol. 8. doi: 10.3389/fcimb.2018.00364 PMC6202873 30406041

[B19] Ptasinska A. Whalley C. Bosworth A. Poxon C. Bryer C. Machin N.. (2021). Diagnostic accuracy of loop-mediated isothermal amplification coupled to nanopore sequencing (LamPORE) for the detection of SARS-CoV-2 infection at scale in symptomatic and asymptomatic populations. Clin. Microbiol. Infect. 27 (9), 1348.e1–1348.e7. doi: 10.1016/j.cmi.2021.04.008 33901668 PMC8064897

[B20] Rui-Chen L. Chang-Qiang Z. Chun-Hui W. Le-le A. Heng L. Bing Z.. (2020). Genetic diversity and population structure of aedes aegypti after massive vector control for dengue fever prevention in yunnan border areas. Sci. Rep. 10 (1), 12731. doi: 10.1038/s41598-020-69668-7 32728176 PMC7391764

[B21] Sarah T. Khamsing V. Eduard B. Sandie M. Massimiliano B. Béatrice R.. (2022). Bat coronaviruses related to SARS-CoV-2 and infectious for human cells. Nature. 604 (7905), 330–336. doi: 10.1038/s41586-022-04532-4 35172323

[B22] Smriti M. (2022). Dozens of unidentified bat species likely live in Asia - and could host new viruses. Nature. 604 (7904), 21. doi: 10.1038/d41586-022-00776-2 35352040

[B23] Susanna K. P. L. Syed S. A. Hazel C. Y. Kenneth S. M. L. Rachel Y. Y. F. Toni Y. C. C.. (2016). Identification and interspecies transmission of a novel bocaparvovirus among different bat species in China. J. Gen. Virol. 97, 12, 3345–3358. doi: 10.1099/jgv.0.000645 27902362

[B24] Susanna K. P. L. Syed S. A. Hoi-Wah T. Hazel C. Y. Kenneth S. M. L. Rachel Y. Y. F. (2017). Bats host diverse parvoviruses as possible origin of mammalian dependoparvoviruses and source for bat-swine interspecies transmission. J. Gen. Virol. 98 (12), 3046–3059. doi: 10.1099/jgv.0.000969 29106348

