# Supplementary figures and images for: Rhinolophus sinicus virome revealed multiple novel mosquito-borne zoonotic viruses
Source: Front Cell Infect Microbiol. 2022 Oct 11;12:960507. doi: 10.3389/fcimb.2022.960507 (PMC9592836; doi:10.3389/fcimb.2022.960507)

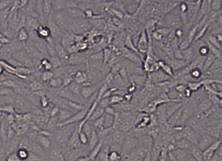

Supplement: Supplementary file 1 [file Image_1.jpeg]

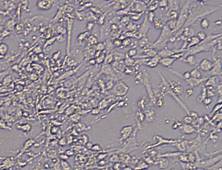

Supplement: Supplementary file 2 [file Image_2.jpeg]

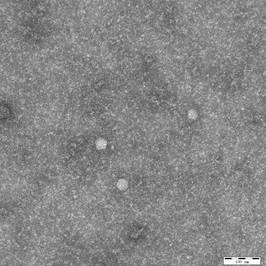

Supplement: Supplementary file 3 [file Image_3.jpeg]

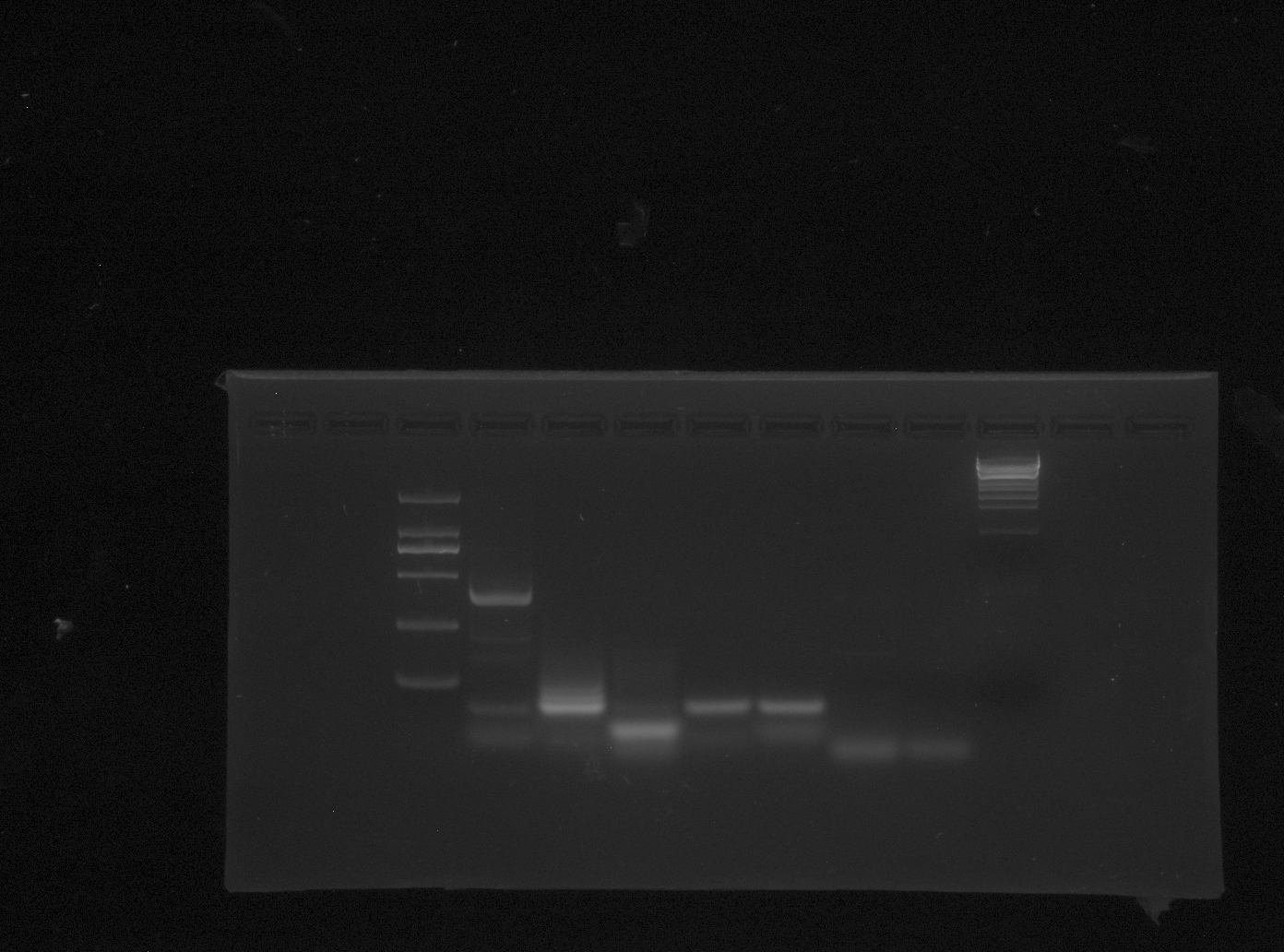

Supplement: Supplementary file 4 [file Image_4.jpeg]

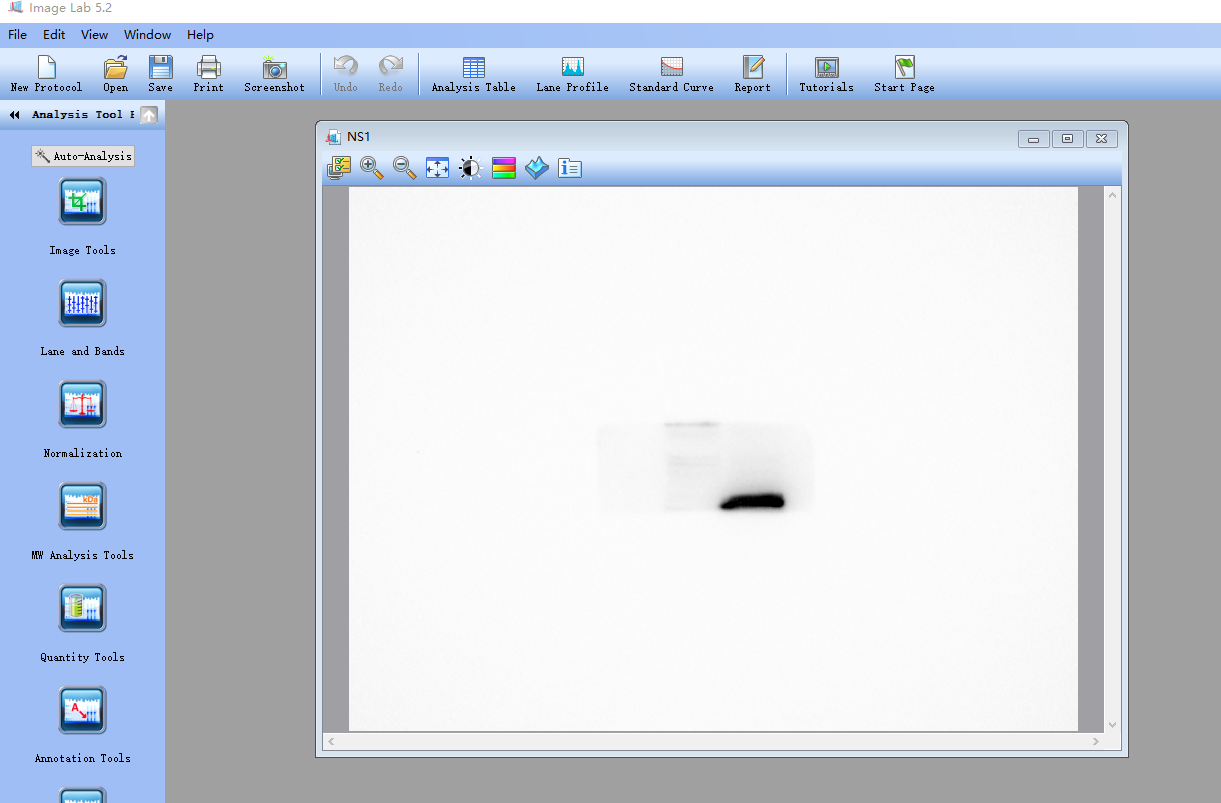

Supplement: Supplementary file 5 [file Image_5.png]

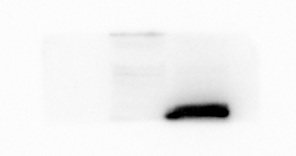

Supplement: Supplementary file 6 [file Image_6.png]

## Slide 1
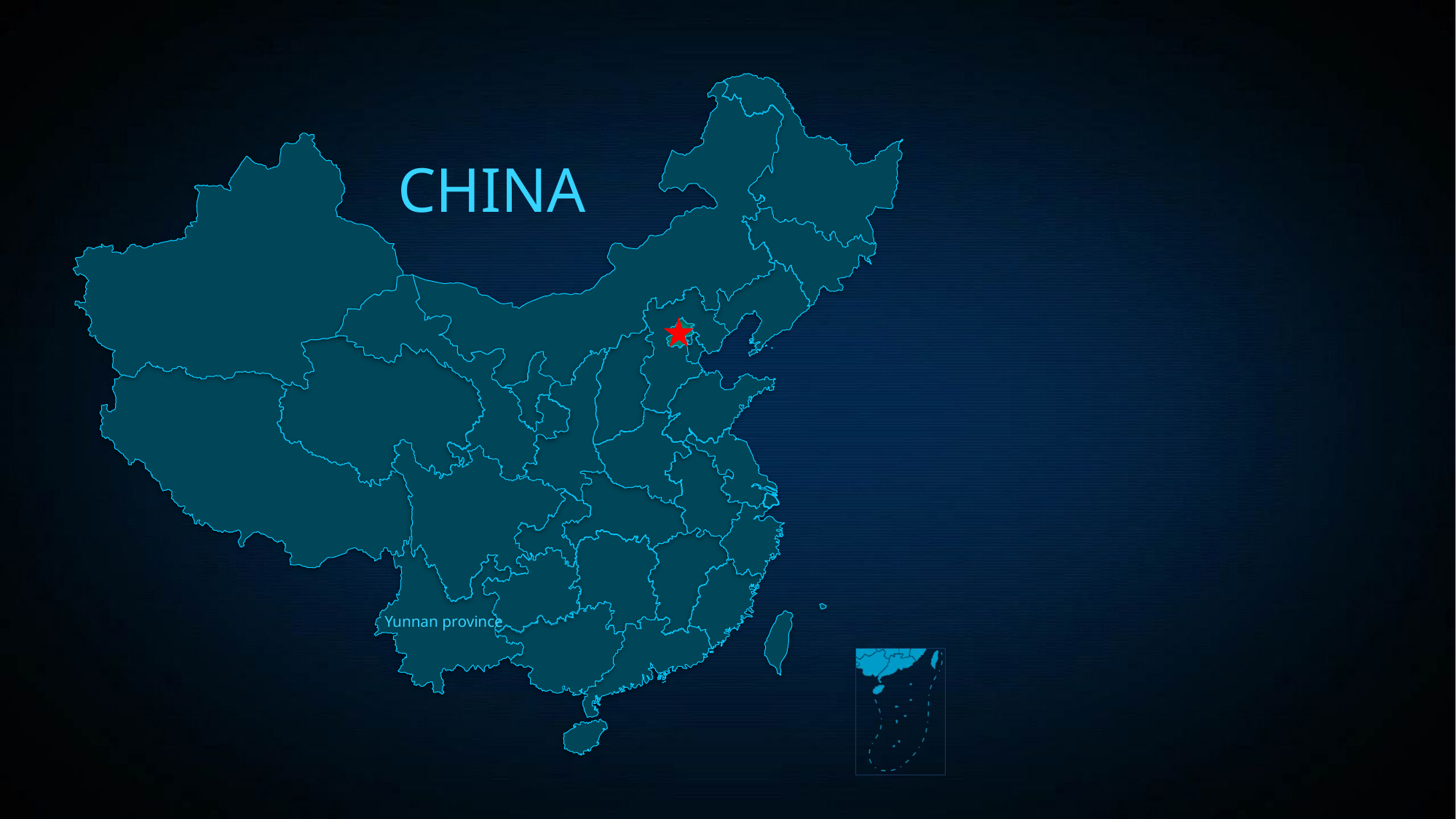

CHINA
Yunnan province

## Slide 2
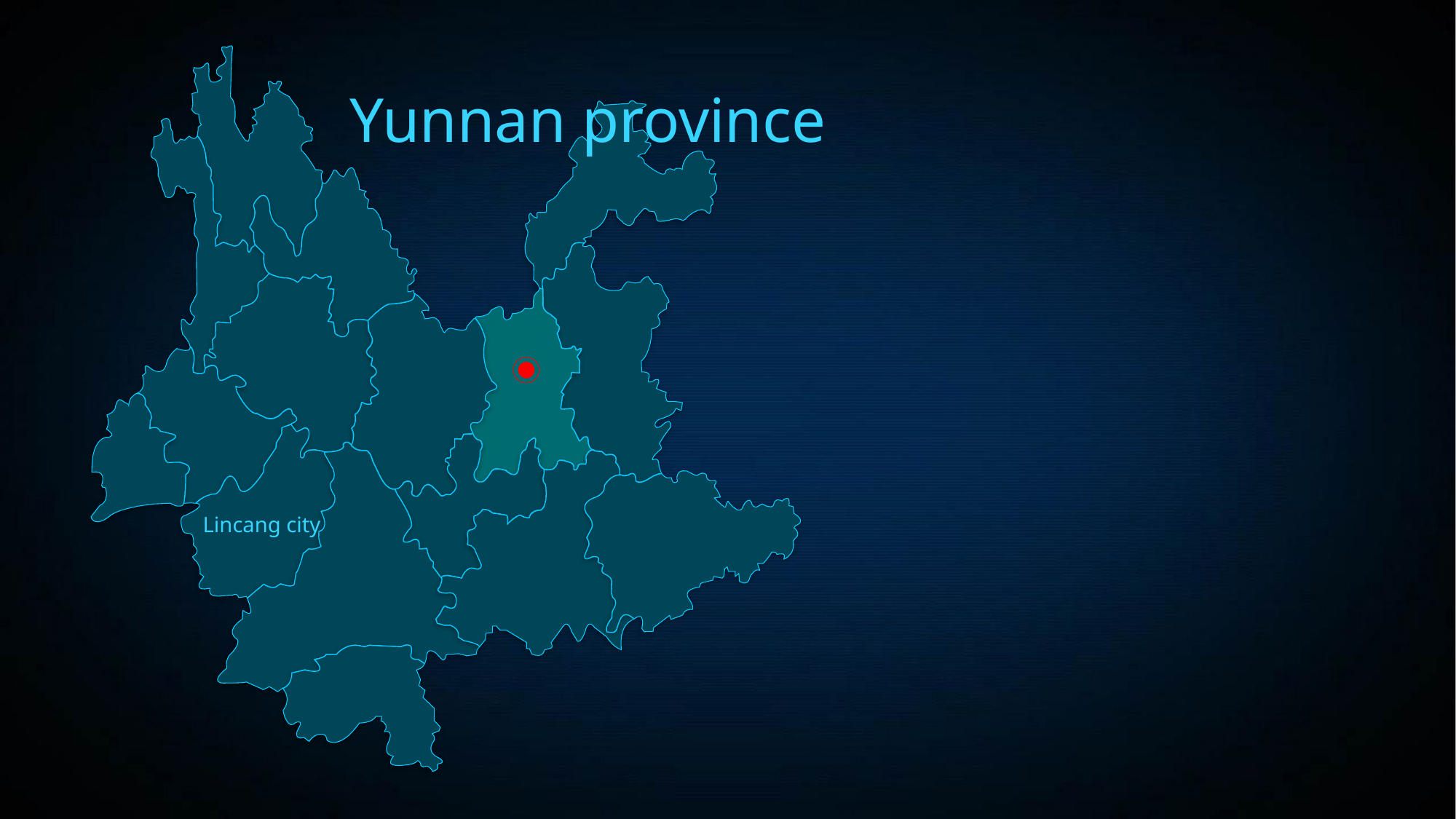

Yunnan province
Lincang city

Supplement: Supplementary file 7 [file Presentation_1.pptx]
